# Ginger relieves intestinal hypersensitivity of diarrhea predominant irritable bowel syndrome by inhibiting proinflammatory reaction

**DOI:** 10.1186/s12906-020-03059-3

**Published:** 2020-09-14

**Authors:** Changrong Zhang, Yongquan Huang, Peiwu Li, Xinlin Chen, Fengbin Liu, Qiuke Hou

**Affiliations:** 1 Graduate School, Guangzhou University of Chinese Medicine, Guangzhou, China; 2 Department of Orthopedics, The Second Affiliated Hospital of Guangzhou University of Chinese Medicine, Guangzhou, China; 3 Department of Gastroenterology, The First Affiliated Hospital of Guangzhou University of Chinese Medicine, Guangzhou, Guangdong 510405 P.R. China; 4 Department of Preventive Medicine and Health Statistics, Guangzhou University of Chinese Medicine, Guangzhou, Guangdong China; 5 School of Chinese Medicine, Hong Kong Baptist University, Hong Kong, China

**Keywords:** Diarrhea predominant irritable bowel syndrome, Ginger, 6-gingerol, Intestinal hypersensitivity, NF-κB pathway

## Abstract

**Background:**

Ginger or ginger extracts have been used in traditional medicine relieve pain caused by diarrhea predominant irritable bowel syndrome (IBS-D), but few data exists about its effectiveness. This present study was to validate the effect of ginger on visceral pain, and to further explore the possible underlying mechanism by which ginger is used to relieve IBS-D intestinal hypersensitivity.

**Methods:**

First, the IBS-D rat model was established by chemical stimulation and acute and chronic pressure stimulation. Then, different dose of ginger were administrated to IBS-D rats and evaluate the defecation frequency, fecal water content (FWC) and abdominal withdrawal reflex (AWR) scores in IBS-D rats. Further, the IBS-D rats were sacrificed to collecte the colonic tissues to evaluate the effect of ginger administration on its pathology and changes of pro-inflammatory factors, and changes of NF-κB pathway. Second, the ginger was taken to HPLC analysis and 6-gingerol was choosen to further experiment. Then, IBS-D rats were treated with different dose of 6-gingerol, and the behavioral evaluation were to evaluate the effect of 6-gingerol on IBS-D rats. Further, colonic epithelial cells (CECs) were collectted and to evaluate the effect of 6-gingerol on the expression of inflammatory factors and changes of NF-κB pathway.

**Results:**

The IBS-D rat model was successfully established by chemical stimulation and acute and chronic pressure stimulation. And ginger treatment significantly reduced the defecation frequency, fecal water content and AWR scores in IBS-D rats. Histopathological analysis showed that ginger treatment can significantly reduce colonic edema and promote the recovery of inflammation in IBS-D rats, and the effect is equivalent to rifaximin. Elisa and RT-qPCR showed that ginger inhibited the expression of proinflammatory factors (TNF-α, IL-6, iNOS) in IBS-D rats. Western blot showed IkBα was up-regulated while p-p65 was inhibited under ginger treatment. HPLC analysis showed that 6-gingerol was the main component of ginger, which could improve clinical symptoms in IBS-D rats. Western blot and RT-qPCR showed that 6-gingerol inhibited the expression of proinflammatory factors (TNF-α, IL-6, iNOS) in CECs, and inhibition of IκBα degradation and phosphorylation of p65 involved in NF-κB pathway.

**Conclusion:**

Ginger and ginger extract could relieve intestinal hypersensitivity of IBS-D by inhibiting proinflammatory response.

## Background

Irritable bowel syndrome (IBS) is a chronic gastrointestinal disorder characterized by recurrent abdominal pain related to a change in bowel habit or defecation frenquency [1]. Diarrhea-predominant irritable bowel syndrome (IBS-D) is generally reported as the most common subtype (28–46% of all IBS) [2]. IBS-D patients have reduced quality of life and may have poorer mood when in pain. Although most of the symptoms of IBS-D will disappear without the use of drugs, patients with severe abdominal pain need to consult a physician for medical treatment [3].

The therapeutic effect of IBS-D is limited, about 40% of patients use alternative drugs to treat abdominal pain, bloating and other symptoms [4]. A randomized, double-blind, placebo-controlled trial have shown that rifaximin treatment is effective and well tolerated in IBS-D, which could relieve IBS-D symptoms, including bloating, abdominal pain, and loose or watery stools [5]. However, the failure rate to relieve IBS-D abdominal pain by rifaximin treatment is between 20 and 25% [6]. The most popular alternative medicine is ginger in a large study of 1012 patients with IBS-D [7]. Ginger, a traditional Chinese medicine, has been widely used for over 2500 years as an anti-inflammatory agent in musculoskeletal disorders [8]. Ginger is generally regarded as safe food by the US Food and Drug Administration, and can be exempted from pre-market review, approval and pre-market clinical testing (http://www.fda.gov/Food/IngredientsPackagingLabeling/GRAS/ucm2006850.htm). There is no records of severe side effects or drug interactions in the Germany’s Commission E Monograph [9]. Ginger root is the rhizome of the *Zingiber officinale* Roscoe, which usually contains 1–3% oil. The dosage of ginger is usually standardized according to the curcumin content, which is believed to have antibacterial effects [10], analgesics [11], antiemetics [12] and other physiological effects. In Micromedex (an evidence-based clinical reference tool for hospitals and physicians; www.micromedex.com) ginger is classified as a broad-spectrum antiemetic drug, which can effectively treat nausea and pregnancy-related vomiting and surgery [13, 14]. Studies have found that ginger can affect pain and bowel movements, which suggests that ginger may help reduce pain and stool changes in IBS-D [15, 16]. Given the known gastrointestinal effects of ginger and its widespread use and low cost, it should be tested as a potential treatment in patients with IBS.

Further, the severity of IBS-D with abdominal pain is directly proportional to the amounts of prostaglandin E2 (PGE2) released [17, 18]. A key step involving in PGE2 production is the conversion of arachidonic acid to prostanoids catalyzed by cyclooxygenase-1 and-2 (COX-1,COX-2) [19]. Previous study have shown that 6-gingerol can inhibit the production of PGE2 and inflammation [20].

Though ginger is the most commonly used herbal medicines for IBS, there is few data exists about its effectiveness and underlying mechanisms. In order to clarify the mechanism of ginger alleviating abdominal pain in patients with IBS-D, we investigated the effect of ginger on an IBS-D rats model and IBS-D rats colonic epithelial cells. To explore the role of ginger in relievesrelieving intestinal hypersensitivity of IBS-D rats and improve clinical symptoms by prevent pro-inflammatory.

## Methods

### Reagents and antibodies

Acetic acid were purchased from Beijing Chemical Reagent Company (Beijing, China). Rifaximin (H20040042) was purchased from Sichuan Star Pharmaceutical Co., Ltd. (Sichuan, China). Phosphate-buffered saline (PBS) was purchased from Shanghai Suobao Biotechnology Co., Ltd. (Shanghai, China). 6-gingerol (purity≥98%) was purchased from Aladdin (Shanghai, China). 8-gingerol (purity≥98%) was purchased from Shanghai Standard Technology Co.,Ltd. (Shanghai, China). Antibodies of anti-IkBα (#9242 L, Cell Signaling, Shanghai, China), anti-Phospho-p65 of the NF-kB (AN371, Beyotime Biotechnology, Shanghai, China), anti-p65 of NF-kB (sc-109, Santa Cruz Biotechnology, Shanghai, China), and anti-β-actin (sc-47,778, Santa Cruz Biotechnology, Shanghai, China) were purchased commercially. Horse radish peroxidase-conjugated secondary antibodies were purchased from Sigma-Aldrich (Shanghai, China). M-MLV(H-) Reverse Transcriptase (R021–01), 2 × Taq Plus Master Mix (P211–01) and ChamQ Universal SYBR qPCR Master Mix(Q711–02) were purchased from Santa Cruz Biotechnology (Dallas, TX, USA).

### Ginger preparation

Fresh and dried ginger (*Zingiber officinale Roscoe)* were purchased from Guangdong Provincial Hospital of Traditional Chinese Medicine. For animal studies, air-dried dried ginger rhizomes samples were ground into 200-mesh powder. Fine powder was suspended in distilled water and delivered by oral administration. Fresh ginger was ground with a food processor. The juice was then collected after removal of cellular debris by centrifugation at 5000×g for 5 mins at room temperature. The liquid was filtered and used as ginger extract for cell culture studies. The property of the extract was profiled by HPLC analysis.

### Animals

Fifty male Specific pathogen-free (6–8 weeks old, 18–220 g) rats were purchased from Laboratory Animal Center of Guangzhou University of Chinese Medicine (Facility approval# SYXK2018–0044). All rats were housed in a vivarium with a controlled environment and provided with food and water ad libitum. All experiments were performed in accordance with Guidelines for the Care and Use of Laboratory Animals and relevant regulations formulated by Guangdong Provincial Committee for the Care and Use of Laboratory Animals.

### IBS-D rats model construction and treatment

The rat model of IBS-D was established essentially as we previously described [21]. Model group were treated with intracolonic instillation of 1 mL 4% acetic acid at 8 cm proximal to the anus for 30s after anesthetized using 2% (0.04 mL/10 g) pentobarbital (intraperitoneal injection). Then, 1 mL PBS was instilled to dilute the acetic acid and flush the colon. The control group was handled identically, except that 1 mL saline was instilled instead of 4% acetic acid. The rats were left to recover from colitis for 7 days. The model group were then subjected unpredictable chronic stress for 3 weeks, followed by 7 days of rest and 1 h of acute restraint stress on day 28. Food (but not water) was removed 10 h prior to the acute restraint stress exposure. The unpredictable chronic stress were as we previously described [22]. Afer model construction, the model group were randomly grouped (10 rats per group) and treated with normal saline, rifaximin (50 mg/kg), ginger (50 mg/kg or 100 mg/kg) by gastric gavage. For 6-gingerol, intraperitoneal injection was applied on rats with a dose of 10 mg/kg or 30 mg/kg (10 rats per group). Before and after 7 days treatment, all rats were subjected to behavioral evaluation and then the rats were sacrificed by cervical dislocation to obtain the distal colon for future experiments.

### Defecation frequency and fecal water content (FWC)

As we previously described [23], the rats were placed in metabolic cages for 24 h with free access to rodent chow and water. Defecation frequency and FWC were used to estimate the colonic motility and sensibility. The defecation frequency was examined by counting the number of fecal pellets for a period of 1 h. The stool was weighed (m0) after collection, and the stool was then weighed again (m1) after it was dried in the oven. FWC was calculated as (m0 – m1)/m0.

### Abdominal withdrawal reflex (AWR) scores

As we previously described [23], AWR scores were used to quantify the colorectal distension (CRD). Simply, the rats were placed in small a cubicle and adapt for 15 min. Then a balloon dilator (2 mm in diameter) was vaseline-coated and inserted into the descending colon which located at 8 cm proximal to the anus, then water was injected to the balloon, leading to CRD. The AWR score was measured by two independent observers using a double-blind method based on the following criteria: 0 points: no behavioral response to CRD; 1 point: simple head movement, then no movement; 2 points: abdominal muscle contraction; 3 points: abdominal rise; and 4 points: back arch and pelvis ascending. When the AWR score was 3, the amount of injected water calculated as the degree of CRD. The experiment was repeated in triplicate to obtain an average value.

### Pathology analysis

After the rats were sacrificed, the distal colon was harvested. For pathological analysis, the colon tissues were excised, weighed, and fixed in a tissue fixative buffer, or snap frozen in liquid nitrogen for RNA isolation. Fixed tissues were dehydrated with 4% paraformaldehyde and then embedded in melted paraffin. The colon tissues were then tailored into 5 μm-thick sections and stained with HE for morphological evaluations. The stained slices were then subsequently observed and photographed with microscope (Olympus BX53) equipped with a camera. The stained slices were then subsequently observed and photographed with microscope (Olympus BX53) equipped with a camera.

### RNA isolation, reverse transcription and real-time qPCR

Colon tissues or cultured cells were used to extract total RNA using TRIzol^®^ reagent. The RNA concentrations were determined using NanoDrop 2000. 1 mg RNA was reverse transcribed with M-MLV(H-)Reverse Transcriptase. Real-time PCR was performed on ABI 7300 Real Time PCR System (Applied Biosystems) using ChamQ Universal SYBR qPCR Master Mix. The data were analyzed using the 2^-ΔΔCt^method to obtain relative abundance (Schmittgen&Livak, 2008). The GAPDH Ct level was used as an internal control for normalization. The primer sequences for each gene are listed in Table 1.

**Table 1:** Primers used for gene induction studies

Gene name	Accession number	Primer pairs (forward and reverse)	Product size (bp)
TNF-α	NM_013693	F: 5′-CTGAACTTCGGGGTGATCGG R: 5′-GGCTTGTCACTCGAATTTTGAGA	122
IL-6	NM_001314054	F: 5′- CTGCAAGAGACTTCCATCCAG R: 5′- AGTGGTATAGACAGGTCTGTTGG	131
iNOS	NM_010927	F: 5′- CAGATCGAGCCCTGGAAGAC R: 5′- CTGGTCCATGCAGACAACCT	249
GAPDH	NM_001289726	F: 5′- AGGTCGGTGTGAACGGATTTG R: 5′- GGGGTCGTTGATGGCAACA	95

### Cell cultures and treatment

As we previously described [21], the distal colons obtained from the model group were cut into small pieces, washed and digested with 0.1% collagenase I and hyaluronidase for 25 mins at 37 °C to separate colonic epithelial cell (CECs). After digestion, the supernatant was transferred into a new tube and dulbecco’s modified eagle medium (DMEM) was added. After centrifuging 3 times, cells were cultured in DMEM solution containing 100 mL/L fetal bovine serum in a CO_2_ incubator with a saturated humidity at 37 °C. Fibroblasts were removed using phase difference digestion and adherence. When 80–90% of the cells were adherent to culture plates, 10, 50, 100, 200 μg/mL ginger extract and 1, 5, 10, 50 μM 6-gingerol were used to intervene CECs.

### Western blotting

As we previously described [21–23], total cell lysates were prepared using lysis buffer with 1% NP-40 and SDS-PAGE protein sample loading buffer supplemented with a cocktail of protease inhibitors. Proteins were separated by SDS-PAGE and transferred to polyvinylidene fluoride membranes, then incubated with their specific antibodies at 4 °C overnight. β-actin was used as a loading control.

### HPLC analysis

The component of the ginger was profiled by HPLC analysis. The ginger powder and ginger extract were soaked in methanol for 48 h and followed by filtration to obtain ginger methanolic extract. Chromatographic separation of the ginger compounds was performed using HPLC (Alliance 2695, Waters, USA) on an Ultimate XB-C18 column (250 × 4.6 mm, 5 μm, Welch, China). The mobile phase was composed of 0.1% phosphoric acid in water (v/v, A) and acetonitrile (B), and a gradient elution program with increasing percentage of solvent B was used. The flow rate was 1.0 mL/min with the UV detector set at 282 nm. Commercially purchased 6-gingerol and 8-gingerol were used as standards.

### Statistical analysis

All measurement data conform to the normal distribution and were expressed as mean ± SEM. All collected data were analyzed using SPSS 21.0 (SPSS Inc., Chicago, IL, USA) and graphic display using GraphPad Prism 6 software (GraphPad, USA). Paired t-test analysis was used for comparison before and after treatment within the group. Statistical differences between groups were assessed by the one-way analysis of variance (ANOVA) in multiple comparisons. *P* value of 0.05 or less was considered as significant.

## Results

### Ginger ameliorated clinical symptoms in IBS-D rats

**Fig. 1:**
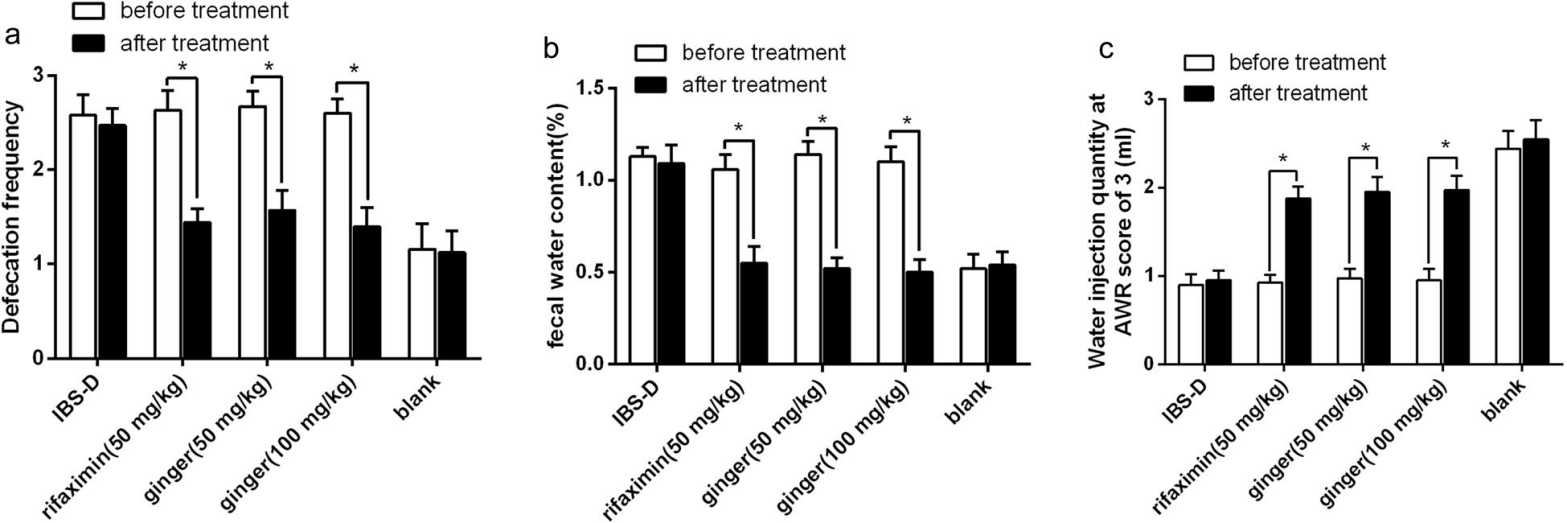
Effects of ginger on defecation frequency, FWC and CRD in IBS-D rats. **a**. Ginger treatment decreased defecation frequency in IBS-D rats. **b**. Ginger treatment decreased FWC in IBS-D rats. **c**. Ginger treatment increased CRD in IBS-D rats. *P < 0.05, when compared to before treatment

Defecation frequency and FWC were significantly increased, while CRD was significantly decreased in IBS-D rats when compared to the blank group. After medication, the defecation frequency and FWC were significantly decreased when compared to the IBS-D model group (*P* < 0.05), while with no difference between rifaximin and ginger (50 or 100 mg/kg) group (Fig. 1a & b). The CRD in ginger (50 or 100 mg/kg) group and rifaximin group were remarkably increased when compared to the IBS-D model group (*P* < 0.05), and no difference between rifaximin and ginger (50 or 100 mg/kg) group (Fig. 1c). These results strongly supported that the successful establishment of IBS-D rat model and ginger ameliorated clinical symptoms in IBS-D rats significantly.

### Ginger reduced colon pathological edema and proinflammatory gene expression

**Fig. 2:**
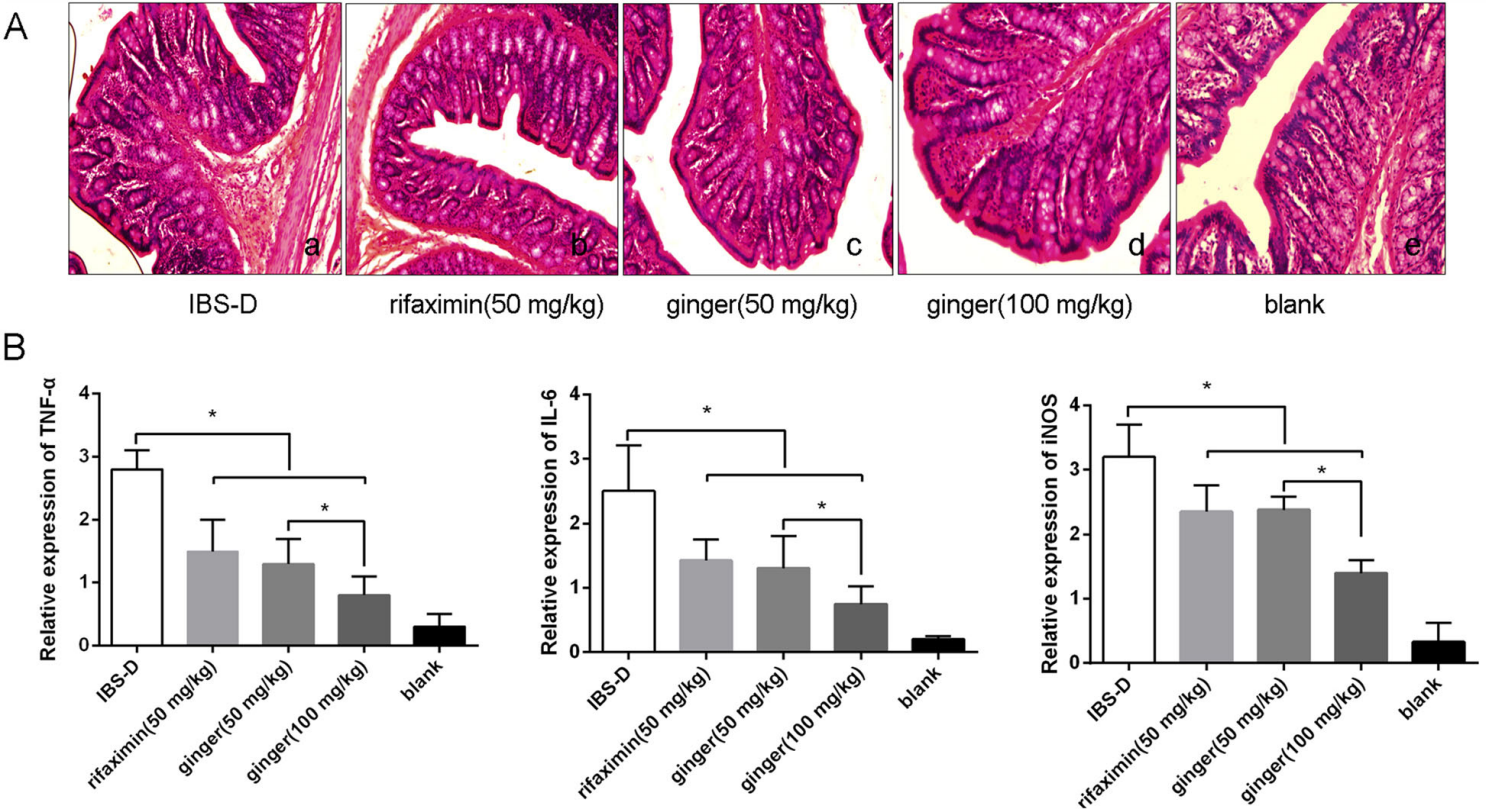
Ginger treatment reduced pathological edema and proinflammatory gene expression. **A**. HE stained colonic epithelial tissues. **a**. IBS-D model group, **b**. rifaximin (50 mg/kg, po), **c**. ginger at 50 mg/kg (po), **d**. ginger at 100 mg/kg (po), **e**. blank group (normal rats). **B**. Ginger treatment reduced proinflammatory gene expression in the colonic epithelial tissues, detected by RT-qPCR. ^#^*P* < 0.05 indicates significant differences compared to the IBS-D model group. *P < 0.05 indicates significant differences between ginger groups (50 and 100 mg/kg)

We performed histological staining to examine whether ginger had an effect on colonic microinflammation. The intestinal mucosal barrier in the normal rats (blank group) showed integrity mediated by CECs, where as CECs in the IBS-D rats displayed significant edema. As shown in Fig. 2A, the colon tissues in IBS-D model rats had obvious alterations in intestinal mucosa epithelium, including impaired intestinal mucosal integrity and the influx of inflammatory cells were increased. Histological analysis of the rat colonic tissues revealed a significant reduction in granulocytes and CECs edema after the administration of ginger (50 or 100 mg/kg) and rifaximin group, compared to the IBS-D model group and blank group. There was increased monocytic cell infiltrates in IBS-D rats. The intestinal mucosal barrier integrity of mucosal layers were were repaired after oral administration of ginger (50 or 100 mg/kg) and rifaximin, and there was no significant difference between ginger (50 or 100 mg/kg) and rifaximin groups. Similar to those in rifaximin-treated group, the structure of the intestinal mucosa epithelium from ginger (50 or 100 mg/kg) treated groups remained relatively intact as the blank group. This result strongly suggested that ginger could significantly repair intestinal mucosal integrity and promote histological recovery in IBS-D rats.

The inhibition on inflammation was confirmed by reduced expression of proinflammatory genes in the colon tissues. As shown in Fig. 2B, compared with the IBS-D group, the expression of TNF-α, IL-6 and iNOS in the ginger group was significantly inhibited. In addition, the ginger group at a dose of 100 mg/kg had better inflammation inhibition than the ginger group at a dose of 50 mg/kg. These results indicate that ginger can inhibit the proinflammatory response, which may be related to suppression of intestinal hypersensitivity in IBS-D.

### Ginger extract inhibited NF-κB activation and proinflammatory gene expression in IBS-D rats CECs

**Fig. 3:**
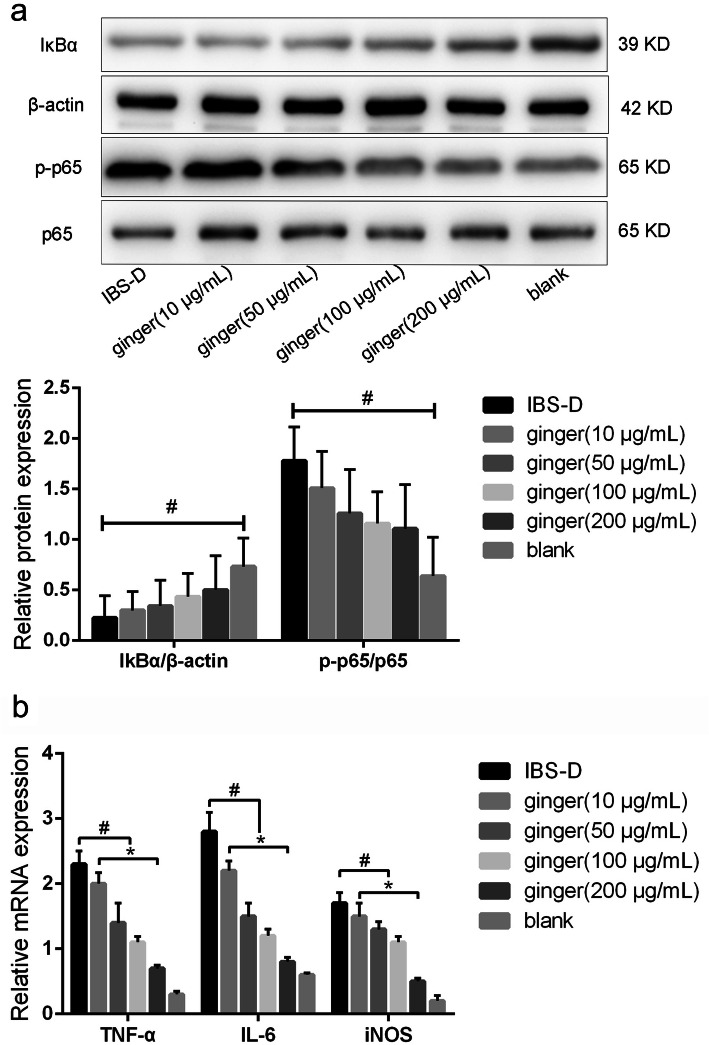
Ginger extract inhibited NF-κB activation and proinflammatory gene expression in IBS-D rats CECs. **a**. Western blot showed ginger extract inhibited NF-κB activation in IBS-D rats CECs (up panel). The relative density of IκBα/β-actin, p-p65/p65 were also showed (down panel). **b**. RT-qPCR showed ginger extract inhibited proinflammatory gene expression in IBS-D rats CECs. ^#^*P* < 0.05 indicates significant differences between IBS-D rats model group and ginger groups (10, 50, 100 and 200 μg/mL). *P < 0.05 indicates significant differences between ginger groups (10, 50, 100 and 200 μg/mL)

To further explore the relationship between the relief effect of ginger on IBS-D and proinflammatory response, we focused on the activation pathway of proinflammatory. In this regard, we used IBS-D rats CECs to determine ginger extract wether against NF-κB pathway activation. Detected by western blot, ginger inhibited the degradation of IκBα and excessive phosphorylation of p65, a functional subunit of NF-κB complex (Fig. 3a, up panel). The relative density of IκBα/β-actin, p-p65/p65 were also showed (Fig. 3a, down panel). This inhibitory effect was also confirmed at mRNA levels in IBS-D rats CECs. As shown in Fig. 3b the CECs from the IBS-D model group significantly increased TNF-α, IL-6 and iNOS mRNA production. Treatment with ginger extract dose-dependently suppressed TNF-α, IL-6 and iNOS gene expression in CECs. These results indicated that ginger extract suppressed proinflammatory response by inhibition of NF-κB pathway activation.

### HPLC profiling of ginger and effect of 6-gingerol on clinical symptoms in IBS-D rats

**Fig. 4:**
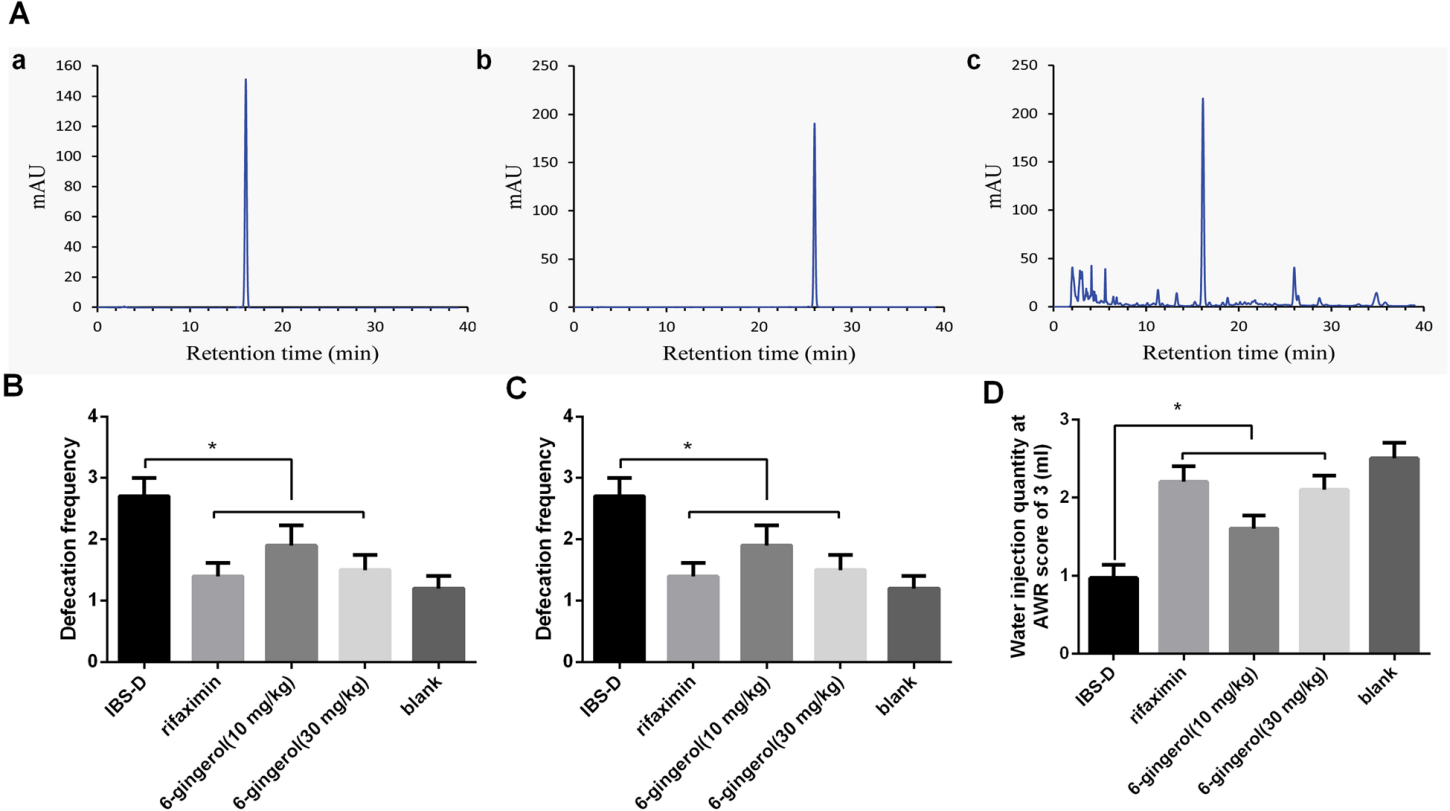
HPLC profiling of ginger and 6-gingerol on clinical symptoms in IBS-D rats. **A**. HPLC profiling of ginger extract. **a**. 6-gingerol, **b**. 8-gingerol, **c**. methanolic extract from fresh ginger. **B, C, D**. Effect of 6-gingerol on clinical symptoms in IBS-D rats. ^*^P < 0.05 indicates significant differences between IBS-D rats model group and medication groups (inclouding rifaximin, 6-gingerol (10 or 30 mg/kg))

There are more than 400 different compounds in the ginger extract, with 6-gingerol and 8-gingerol as the major non-volatile, pungent and bioactive principles. We performed an HPLC analysis to identify the presence of gingerols in the extract using commercial 6-and 8-gingerol as standards. As shown in Fig. 4A, 6-gingerol was among the major component in ginger extract. We then investigated whether 6-gingerol was an effective compound for ginger anti-intestinal hypersensitivity activity using behavioral evaluation. As shown in Fig. 4B & C, the defecation frequency and FWC were significantly decreased when compared to the IBS-D model group (*P* < 0.05), while with no difference between rifaximin and 6-gingerol (30 mg/kg) group. The CRD in 6-gingerol (10 or 30 mg/kg) group and rifaximin group were remarkably increased when compared to the IBS-D model group (*P* < 0.05), and no difference between rifaximin and 6-gingerol (30 mg/kg) group (Fig. 4D). This result indicated that 6-gingerol was the effective compound for ginger anti-intestinal hypersensitivity activity in IBS-D rats.

### Effect of 6-gingerol on the NF-κB pathway activation and proinflammatory gene expression in IBS-D rats CECs

**Fig. 5:**
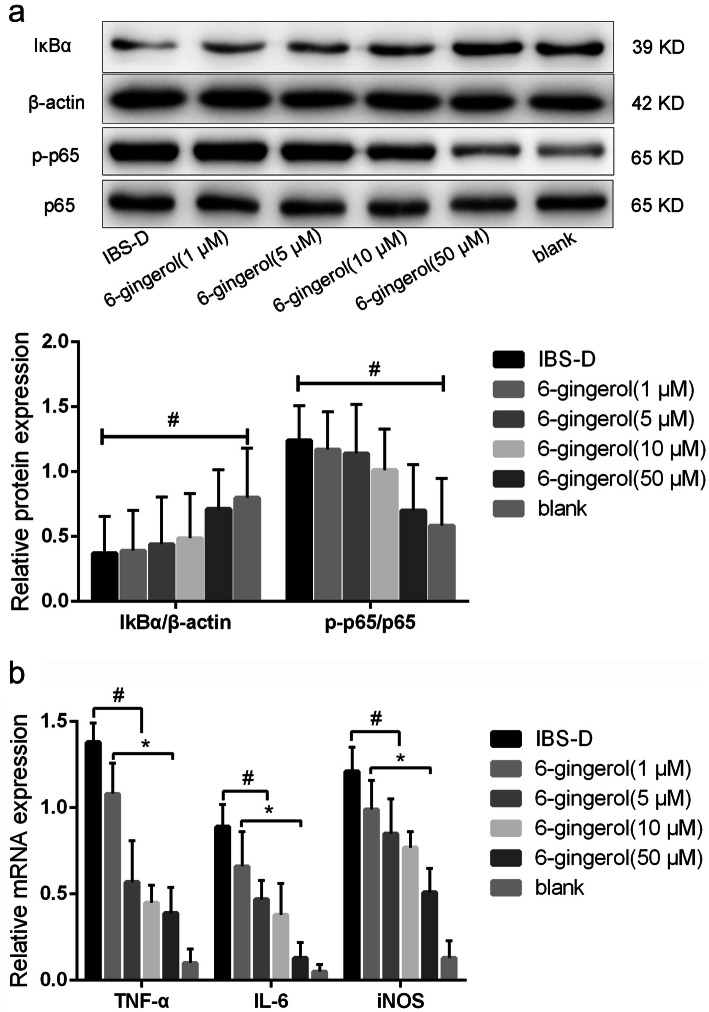
Effect of 6-ginger on NF-κB pathway activation and proinflammatory gene expression in IBS-D rats CECs. **a**. Western blot showed effect of 6-gingerol on NF-κB activation in IBS-D rats CECs (up panel). The relative density of IκBα/β-actin, p-p65/p65 were also showed (down panel). **b**. RT-qPCR showed effect of 6-gingerol on proinflammatory gene expression in IBS-D rats CECs. ^#^*P* < 0.05 indicates significant differences between IBS-D rats model group and 6-gingerol groups (1, 5, 10 and 50 μM). *P < 0.05 indicates significant differences between 6-gingerol groups (1, 5, 10 and 50 μM)

Further we studied the effect of 6-gingerol on the NF-κB pathway activation and proinflammatory gene expression in IBS-D rats CECs. Detected by western blot, 6-gingerol dose-dependently blocked IκBα degradation as well as p65 phosphorylation in IBS-D rats CECs, which indicated that 6-gingerol showed potent inhibitory activity against IBS-D rats CECs NF-κB pathway activation (Fig. 5a, up panel). The relative density of IκBα/β-actin, p-p65/p65 were also showed (Fig. 5a, down panel). Similarly, the 6-gingerol also dose-dependently blocked TNF-α, IL-6, iNOS experssion in IBS-D rats CECs, determined by RT-qPCR (Fig. 5b). This result demonstrated that 6-gingerol, the major component ginger, had strong anti-intestinal hypersensitivity activity and anti-inflammation activity.

## Discussion

IBS is a clinical syndrome characterized by chronic discomfort or pain in abdomen caused by disturbed bowel habit, which can be elaborately categorized into four subtypes [24]. IBS-D is generally reported as the most common subtype (28–46%) of all IBS [2]. IBS-D is a multifactorial disease and many studies have demonstrated that visceral hypersensitivity [25], gut barrier disfunction [26], aberrant microbiota-brain-gut interaction [27] and intestinal motility abnormality [28] are the pivotal participators in the pathogenesis. Conventional medications for IBS-D, such as antidiarrheal, antispasmodic,antibiotics and probiotics, often have limited effects and repeated treatment brings about tremendous socio-economic pressure [29]. Therefore, more effective approaches or agents for the treatment of IBS-D are needed. And the therapeutic focus has naturally shifted to traditional medicine.

As a traditional medicine used for thousands of years, ginger has shown remarkable efficacy in inflammatory diseases. In Micromedex, ginger is classified as a broad spectrum antiemetic, and is effective in treating nausea and vomiting associated with pregnancy and surgery [30, 31]. Ginger is also one of the most commonly used herbal medicines by IBS patients, however, the relevant mechanism is not yet clear. The underlying mechanism for reducing abdominal pain involves the production of PGE2 and a pro-inflammatory response [32]. In this study, we found that ginger can improve intestinal hypersensitivity in the IBS-D rat model, and can relieve the symptoms of IBS-D rats diarrhea and abdominal pain, and accompanied by a reduction in proinflammatory response. These results indicate that the therapeutic effect of ginger on IBS-D may be related to its anti-inflammatory activity, but the specific treatment mechanism needs to be explored.

The NF-κB pathway plays a central role in regulating immune and inflammatory processes and thus becomes the target for developing new therapies of inflammatory diseases [33]. Transcriptional control of the vast majority of genes involved in inflammation requires NF-κB activation [34]. Prior to the activation, NF-κB is tethered by association with IκBα, an inhibitory protein that keeps NF-κB in an inactive state in the cytoplasm [35]. Various stimuli such as proinflammatory factors induces IκBα degradation, which leads to the release of NF-κB. The liberated NF-κB translocates to the nucleus to regulate transcription of certain genes [36]. We showed that ginger extract as well as 6-gingerol, the most abundant and active ingredient in ginger, strongly inhibited IκBα degeneration and p65 phosphorylation. Therefore, it can be considered that ginger extract and 6-gingerol play an important role in anti-inflammatory in the treatment of IBS-D. Administration of 6-gingerol also improved the clinical symptoms of IBS-D rats.

## Conclusion

Our research shows that ginger treatment can significantly reduce the frequency of defecation and colonic edema in IBS-D rats, and can inhibit the expression of pro-inflammatory factors in IBS-D rats. 6-gingerol has a potential inhibitory effect of pro-inflammatory factors release in CEC of IBS-D rats. Both ginger and 6-gingerol can reduce the intestinal allergies of IBS-D by inhibiting the pro-inflammatory response which involved in NF-κB signaling pathway. These findings provide evidence that ginger and 6-gingerol are potential protective agents for IBS-D intestinal hypersensitivity. It is necessary to further study the intestinal barrier protective function of ginger and 6-gingerol.

## Supplementary information

**Additional file 1.**

**Additional file 2.**

## Data Availability

The data used to support the findings of this study are available from the corresponding author upon request.

## Abbreviations

IBS: Irritable bowel syndrome
IBS-D: Diarrhea-predominant irritable bowel syndrome
PGE2: Prostaglandin E2
COX-1,COX-2: cyclooxygenase-1 and-2
HE: Hematoxylin-Eosin
FWC: Fecal water content
CRD: Colorectal distension
CECs: Colonic epithelial cell
DMEM: Dulbecco’s modified eagle medium
