# Supplementary figures and images for: Ginger relieves intestinal hypersensitivity of diarrhea predominant irritable bowel syndrome by inhibiting proinflammatory reaction
Source: BMC Complement Med Ther. 2020 Sep 14;20:279. doi: 10.1186/s12906-020-03059-3 (PMC7489045; doi:10.1186/s12906-020-03059-3)

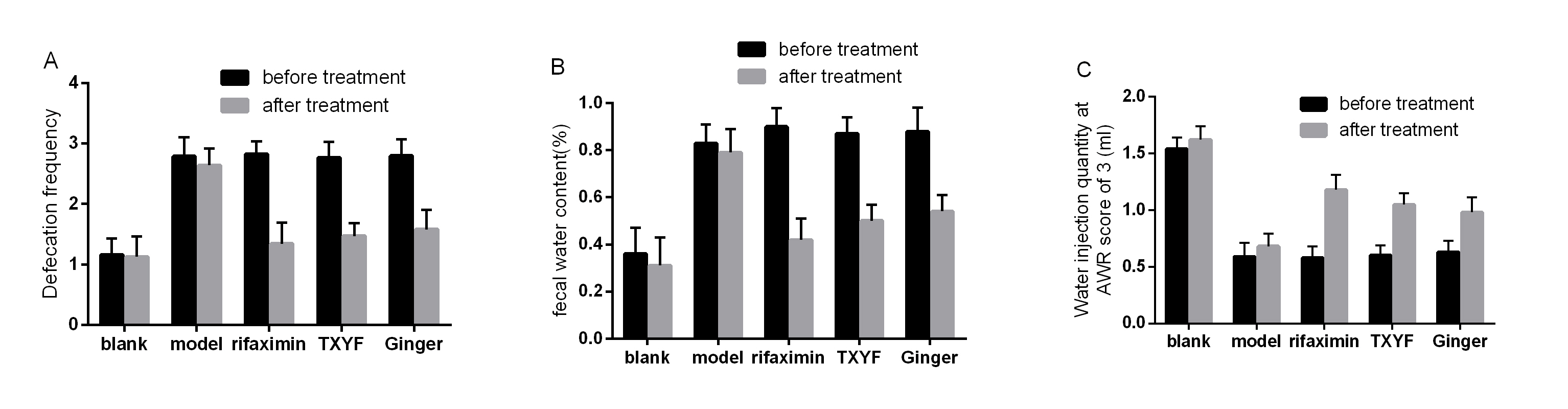

Supplement: Supplementary file 2 — Additional file 2. [file 12906_2020_3059_MOESM2_ESM.jpg]
